# 7 days of L-citrulline supplementation does not improve running performance in the heat whilst in a hypohydrated state

**DOI:** 10.1007/s00421-024-05671-4

**Published:** 2024-12-19

**Authors:** Thomas G. Cable, Mark P. Funnell, Kirsty M. Reynolds, Ella F. Hudson, Heather Z. Macrae, Drusus A. Johnson, Lee Taylor, Liam M. Heaney, Stephen A. Mears, Stephen J. Bailey, Lewis J. James

**Affiliations:** 1 National Centre for Sport and Exercise Medicine, School of Sport, Exercise and Health Sciences, Loughborough University, Loughborough, UK; 2 NIHR Applied Research Collaboration East Midlands, Diabetes Research Centre, University of Leicester, Leicester, UK; 3 Tottenham Hotspur Football Club, Enfield, UK; 4 Diabetes Research Centre, University of Leicester, Leicester, UK; 5 University of Technology Sydney, Sydney, NSW Australia; 6 NIHR Leicester Biomedical Research Centre, University Hospitals of Leicester NHS Trust and the University of Leicester, Leicester, UK

**Keywords:** Endurance, Hydration, Ergogenic aid, Time trial, Thermoregulation

## Abstract

**Purpose:**

7 days L-citrulline supplementation has been reported to improve blood pressure, $\mathop {\text{V}}\limits^{.}$O_2_ kinetics, gastrointestinal (GI) perfusion and endurance cycling performance through increasing arterial blood flow. In situations where blood volume is compromised (e.g., hyperthermia/hypohydration), L-citrulline may improve thermoregulation and exercise performance by redistributing blood flow to aid heat loss and/or muscle function. This study assessed 7 days L-citrulline supplementation on running performance in the heat, whilst mildly hypohydrated.

**Methods:**

13 endurance runners (2 female, 31 ± 8 y, $\mathop {\text{V}}\limits^{.}$O_2_peak 60 ± 6 mL/kg/min) participated in a randomised crossover study with 7 days L-citrulline (CIT; 6 g/d) or placebo (maltodextrin powder; PLA) supplementation. Participants completed a 50 min running ‘preload’ at 65% $\mathop {\text{V}}\limits^{.}$O_2_peak (32 °C, 50% relative humidity) to induce hyperthermia and hypohydration before a 3 km running time trial (TT). Body mass and blood samples were collected at baseline, pre-preload, post-preload and post-TT, whilst core and skin temperature, heart rate and perceptual responses were collected periodically throughout.

**Results:**

TT performance was not different between trials (CIT 865 ± 142 s; PLA 892 ± 154 s; *P* = 0.437). Core and skin temperature and heart rate (*P* ≥ 0.270), hydration (sweat rate, plasma volume, osmolality) indices (*P* ≥ 0.216), GI damage (*P* ≥ 0.260) and perceptual responses (*P* ≥ 0.610) were not different between trials during the preload and TT.

**Conclusions:**

7 days of L-citrulline supplementation had no effect on 3 km running performance in the heat or any effects on thermoregulation or GI damage in trained runners in a hypohydrated state.

**Supplementary Information:**

The online version contains supplementary material available at 10.1007/s00421-024-05671-4.

## Introduction

Exercise increases metabolic rate (energy expenditure) with approximately 75% of this energy turnover appearing as heat production. Heat production rises immediately during exercise and when it exceeds heat loss, there is a rise in body temperature that triggers thermoregulatory responses, such as increased skin blood flow and sweating to attenuate body temperature increases (Périard et al. 2021). During exercise in hot environments, conductive and convective heat loss capacity are reduced, making evaporative heat loss through sweating vital for thermoregulation (Dugas et al. 2009). As these processes require a redistribution of blood flow away from contracting locomotor muscles, prolonged exercise in the heat increases cardiovascular, thermal and perceptual strain, leading to impaired endurance performance compared to exercise in temperate/cool environments (Cheuvront and Kenefick 2014).

The increased sweat rate during exercise in hot environments further challenges thermoregulation as hypohydration is likely to accrue during prolonged endurance exercise, particularly whilst running (Dion et al. 2013). Drinking sufficient fluid to fully, or even partially, replace sweat losses can be difficult during running (James et al. 2019), due to the practical challenges of carrying fluid and the potential for gastrointestinal (GI) discomfort with high fluid ingestion rates (Rollo et al. 2012; Dion et al. 2013). Hypohydration leads to a reduction in total blood volume (i.e., hypovolemia), further compromising cardiovascular function and thermoregulation (James et al. 2019), as well as potentiating GI damage (Costa et al. 2019). In combination, these physiological alterations reduce peak oxygen uptake (Nybo et al.  2001), increase perceived exertion (James et al. 2019), and may compromise running performance (Funnell et al. 2023).

As replacing sweat losses might be particularly problematic with endurance running, especially with greater sweat rates in the heat, strategies that may lessen the negative impact of hyperthermia and/or hypohydration on exercise performance warrant further investigation to minimise the ergolytic effects of heat on endurance running performance. Previous strategies have included pre-exercise hyperhydration (Jardine et al. 2023) and exercise heat acclimation (Lorenzo et al. 2010), which can both independently increase plasma volume and improve endurance performance. However, research suggests that pre-exercise hyperhydration may negatively impact GI comfort during running (Latzka et al. 1998), whilst heat acclimation is not always accessible, due to travel and financial restrictions.

One potential strategy is to increase endothelial nitric oxide synthase (eNOS)-derived nitric oxide (NO) bioavailability in the blood. As NO is a potent vasodilator, this may have potential to increase arterial blood flow for increased muscular work and facilitate endurance performance and/or improve thermoregulation (Endo et al. 1994). NO can modulate cutaneous vascular control and improve thermoregulation at rest (Kellogg et al. 1999), as well as increase GI perfusion (Shah et al. 2004) and reduce GI damage in the heat. Supplementation with inorganic dietary nitrate has been shown to increase eNOS-derived NO (Wylie et al. 2013), but with little influence on endurance performance in hot environments (Amano et al. 2018; Kent et al. 2018; Smith et al. 2019; Fowler et al. 2021).

A separate way of increasing eNOS-derived NO is supplementation with the non-essential amino acid, L-citrulline. Similar to dietary nitrate, L-citrulline supplementation increases NO bioavailability by increasing plasma L-arginine concentrations (Rowlands et al. 2012). This occurs through the CIT-NO pathway (Bailey et al. 2015), which converts L-citrulline to arginosuccinate via argininosuccinate synthase, and then to L-arginine via arginoinosuccinate lyase in the kidney and tissues (Curis et al. 2005). Upon L-arginine entering the blood stream, it is then converted to NO via eNOS (Wijnands et al. 2012). Furthermore, L-citrulline has capacity to increase plasma L-arginine levels to a greater extent than supplementation with L-arginine, since ~ 50% of exogenous L-arginine is catabolised during first pass metabolism (Closs et al. 1997; Schwedhelm et al. 2008). As L-citrulline is not subject to this catabolism, and with its natural conversion to L-arginine, it can augment circulating L-arginine compared to L-arginine supplementation. Using L-citrulline in this scenario may be beneficial, as plasma L-arginine may further increase NO bioavailability compared to dietary nitrate alone (Sandbakk et al. 2015).

Animal models suggest beneficial thermoregulatory effects of increased circulating L-arginine, with core body temperature reductions observed in thermally strained rodents performing running exercise (Wanner et al. 2015) and broilers at rest (Uyanga et al. 2021). In humans, acute L-citrulline dosage increases splanchnic microcirculation during exercise and reduces gastrointestinal permeability (van Wijck et al. 2014). Following 7 days (6 g/day; (Bailey et al. 2015)) and 8 days (2.4 g/day; (Suzuki et al. 2016)) supplementation, L-citrulline can enhance high-intensity endurance performance, although this is not a universal finding (Shanely et al. 2016; Bailey et al. 2016). Therefore, due to the potential beneficial alterations in thermoregulation and cardiovascular function following increased L-arginine bioavailability, L-citrulline supplementation may improve running performance during exercise in the heat, where hypohydration and hypovolemia are likely to accrue.

Therefore, the aim of this study was to investigate the effect of 7 days L-citrulline supplementation on running performance, thermoregulation, and GI responses in the heat, where high sweat rates and reductions in blood volume are likely. It was hypothesised that L-citrulline supplementation would increase 3 km running performance, as well as reducing body temperature and gastrointestinal damage.

## Methodology

*Participants.* Thirteen trained (at least Tier 2; McKay et al. (2021)), non-heat acclimated, endurance runners (11 male, 2 female; mean ± SD: age 31 ± 8 y, stature 1.75 ± 0.06 m, body mass 71.1 ± 10.5 kg, $\mathop {\text{V}}\limits^{.}$O_2_peak 60 ± 6 mL/kg/min) participated in this study, which received ethical approval from the Loughborough University Ethics Approvals (human participants) Sub-Committee (Reference: 2021-4673-4374). Initially, fifteen participants were recruited, but two withdrew following the first experimental visit due to injury unrelated to the study. Participants completed a health screening questionnaire and provided verbal and written informed consent before participating. Participants completed four trials: a preliminary visit, familiarisation trial and two experimental trials at the same time of day (standardised within-participant) in a double blind, placebo-controlled, randomised manner. The familiarisation trial and first experimental trial were separated by ≥ 7 days, and the experimental trials were separated by ≥ 3 weeks, allowing for a 2-week washout period (Ashley et al. 2018) and a further week of supplementation before the second experimental trial. For female participants, trials were separated by the same minimum time frames, but experimental trials also occurred during the same phase of the menstrual or contraception pill cycle (identified via participant confirmation).

*Preliminary testing.* During the first visit, body mass and stature were measured. Running peak oxygen uptake ($\mathop {\text{V}}\limits^{.}$O_2_peak) was determined using a motorised treadmill (HP Cosmos, Nussdorf, Germany). Participants completed four, 4-min stages at progressively increasing speeds until a heart rate of  >160 beat/min was obtained. Expired gas was collected into a Douglas bag during the final min of each stage and analysed for oxygen and carbon dioxide concentrations (Servomex 1400 Gas Analyser, Servomex, Crowborough, UK), volume (Harvard dry gas meter, Harvard Apparatus Ltd, Edenbridge, UK) and temperature (RS Pro digital thermometer, RS Components, Corby, UK). Ambient air was collected concurrently with expired gases to correct $\mathop {\text{V}}\limits^{.}$O_2_ and $\mathop {\text{V}}\limits^{.}$CO_2_ (Betts and Thompson 2012). Following a self-selected rest period (to ensure sufficient recovery), participants ran to volitional exhaustion via a ramp protocol, where speed of the final sub-maximal stage was maintained, with incline increasing by 1% each min until volitional exhaustion. A final expired gas sample was collected during the final min of exercise. These data were then extrapolated using linear regression to determine the speed equivalent to 60–65% of $\mathop {\text{V}}\limits^{.}$O_2_peak for use in subsequent trials. Following a short rest period (~ 5–10 min), participants practiced the 3 km time trial. During the familiarisation trial, participants completed a replication of the experimental trials (outlined below) including all measures except prior supplementation.

*Pretrial standardisation.* In the 24 h preceding their first experimental trial, participants recorded their dietary intake and physical activity and replicated these patterns prior to the second experimental trial. In the 24 h before each trial, participants were asked to refrain from vigorous exercise and alcohol consumption, and were asked to consume a minimum of 40 mL/kg body mass of fluid (any fluid excluding alcoholic drinks) to help ensure euhydration upon arrival to the laboratory (Minshull and James 2013). On the day of testing, participants consumed a standardised pre-trial meal providing 1 g carbohydrate/kg body mass and 7 mL/kg body mass of fluid consisting of cereal bars, orange juice (Tesco PLC, Welwyn Garden City, UK) and tap water 45 min before arrival at the laboratory.

*Supplementation procedures.* Experimental trials were preceded by six consecutive days of supplementation, with the seventh and final dosage taken on the day of experimental trials. All supplementation consisted of 6 g/day L-citrulline (NOW Foods, Bloomingdale, IL, USA) (CIT trial) or 6 g/day maltodextrin (MyProtein, Cheshire, UK) as a placebo (PLA trial). Each daily supplement dose was provided in 12, size 00 vegan capsules (Bulk, Essex, UK). Participants were instructed to consume daily supplements at a similar time of day as their scheduled trial time (outside of mealtimes), with ~ 300 mL of water and within a 5 min period.

During experimental trials, participants consumed the final supplement dose with 300 mL diluted sugar-free squash, with 6 g (CIT) or 0 g (PLA) maltodextrin powder to match acute carbohydrate intake between trials. Experimental supplementation and drinks were administered in a double-blind manner, where an investigator not involved in data collection randomised the supplements and made up the drinks. The timing of this supplementation was in line with previous pharmacokinetic data (Schwedhelm et al. 2008) indicating peak plasma L-arginine ~ 90–100 min after 6 g L-citrulline oral supplementation. The current protocol was designed to illicit peak plasma L-arginine at the beginning of the time trial (Table 1).

**Table 1:** Plasma L-citrulline and L-arginine concentration within each trial. Data presented as median (IQR)

		Baseline	Pre-preload	Post-preload	Post-TT
L-arginine (μmol/L)	CIT	161 (109–209)*	177 (113–238)*	213 (174–250)*	226 (148–302)*
	PLA	95 (72–122)	101 (74–101)	99 (72–113)	104 (71–119)
L-citulline (μmol/L)	CIT	76 (45–103)*	1008 (89–1505)*	1290 (364–1583)*	976 (297–1406)*
	PLA	41 (20–64)	106 (25–67)	205 (26–73)	174 (28-77)

* indicates significant difference to PLA within time point

*Experimental trials.* Participants ingested a radiotelemetry pill (BodyCap, e-Celsius® Performance, Hérouville Saint-Clair, France) 8–10 h prior to arrival to measure body core temperature. Upon arrival, participants provided a total void urine sample, before nude body mass was recorded. Participants were fitted with a wireless heart rate monitor (Polar M400, Polar, Kemple, Finland) and four wireless skin thermistors (iButton DS1922L Thermochron Data Logger; Berkshire; UK) on the right chest, triceps, thigh and calf, for measurement of heart rate and skin temperature respectively, with weighted mean skin temperature calculated (Ramanathan 1964). Thereafter, participants assumed an upright seated position, and after 20 min, venous blood was collected from an antecubital/forearm vein and core and skin temperature recorded. Participants then consumed their final supplement dose and 40 min later, a venous blood sample and urine sample were collected, before nude body mass was measured. Participants then entered the environmental chamber (32 °C and 50% relative humidity) and completed 50 min continuous running at 60–65% $\mathop {\text{V}}\limits^{.}$O_2_peak (preload). During the preload of both trials, participants consumed a small bolus of water (0.5 mL/kg body mass) at 25 and 50 min (total 36 ± 5 mL) to minimise discomfort and to allow hypohydration to accrue during exercise. Upon completion of the preload, participants sat for an immediate venous blood sample, before nude body mass was measured. Participants then completed a 3 km time trial. For the time trial, the treadmill was stationary, and the investigator counted down from 3 (3, 2, 1, GO!), with participants able to control treadmill speed via a controller proximal to their right side. Participants were told that this was ‘a performance test’ and were instructed to ‘complete the 3 km as quickly as possible’ and were blinded to all feedback, except distance completed. The same investigator sat behind the treadmill and verbally notified the participants at each kilometre using standardised wording, with no further interaction between investigator and participant. Upon completion of the time trial, participants exited the chamber and towel dried, before nude body mass was measured, and a urine sample collected. Finally, participants sat for 20 min before a venous blood sample was collected.

*Measures.* Heart rate, core and skin temperature were recorded at baseline, 40 min following supplementation (pre-preload), every 10 min throughout the preload, and every 1 km of the time trial. Expired gas samples were collected at 24–25 and 49–50 min of the preload. Rating of perceived exertion (RPE; (Borg 1982)), GI comfort (Jeukendrup et al. 2000) and thermal sensation (RTS; −10 to + 10 scale; (Lee et al. 2008)) were recorded at baseline, 25 and 50 min of the preload and at the end of the time trial. Local sweat rate was measured via placement of a technical absorbent patch on the lower back (Tegaderm Plus; 3M Health-care, Loughborough, U.K) for the final 10 min of the preload. Sweat patch area was measured, and patches weighed to the nearest 0.0001 g (Sartorius, Germany) pre and post application to determine local sweat rate (Morris et al. 2013).

*Sample analyses.* For each blood sample, 11 mL of blood was drawn, of which 1 mL was dispensed into a tube containing K_2_ EDTA (1.75 mg/mL; Teklab, Durham, UK) and used to measure haemoglobin concentration via the cyanmethemoglobin method in duplicate and haematocrit via microcentrifugation in triplicate. These values were used to determine changes in blood, red cell, and plasma volume relative to baseline (Dill and Costill 1974). A further 10 mL of blood was dispensed into Lithium Heparin tubes (0.25 mg/mL; Sarstedt AG & CO., Nümbrecht, Germany), centrifuged (2500 *g*, 20 min, 4 °C) and plasma was stored at −80 °C until analysed for osmolality via freezing-point depression (Gonotec Osmomat 030 Cryoscopic Osmometer; Gonotec, Germany; CV 0.3%), intestinal fatty acid binding protein (I-FABP) via enzyme linked immunosorbent assay (ELISA; R&D Systems, Minneapolis, USA; CV 8.3%), and L-citrulline and L-arginine concentrations via liquid chromatography-tandem mass spectrometry using an adapted protocol previously validated by Shin et al. (2015) [(CV 4.0% L-arginine and 7.5% L-citrulline); see supplementary material for full method].

*Statistical analyses.* Sample size was determined using G*Power (Version 3.1.9.2) and previous data from our laboratory for the same 3 km performance test (Funnell et al. 2023). It was estimated that nine participants would be sufficient to detect a 5% difference in 3 km running time trial performance with a statistical power of 0.8, and 13 participants with a statistical power of 0.95. Data were analysed using SPSS (version 28; SPSS) and were checked for normality of distribution using a Shapiro–Wilk test. Data containing two factors were analysed using a two-way (trial*time) repeated measures ANOVA. If the assumption of sphericity was violated, the Greenhouse–Geisser estimate was used to correct the degrees of freedom. If significant, interaction and trial effects were followed by a post hoc paired *t*-test or Wilcoxon signed rank test, depending on normality of data. Familywise error was controlled using a Holm–Bonferroni correction. Data containing one factor (baseline data, time trial performance, local sweat rate) were analysed via paired *t*-test or Wilcoxon signed rank test, as appropriate. Hedge’s G effect sizes (*g*) were calculated and interpreted as small (0.2–0.49), medium (0.5–0.79) and large (≥ 0.8). Data sets were accepted as statistically different when *P* ≤ 0.05. All data were screened for outliers, with one outlier observed for L-citrulline and L-arginine according to the ± 3 MAD method (Leys et al. 2013). Plasma L-citrulline values during exercise for the outlier were > 21 standard deviations above the mean of the other 12 participants, therefore, this was removed from the results. Data are presented as mean ± standard deviation, unless otherwise specified.

## Results

### Trial conditions

#### Pre-trial measures

Pre-trial body mass (CIT 71.2 ± 10.5 kg; PLA 70.9 ± 11.0 kg; *P* = 0.281), plasma osmolality (CIT 287 ± 5 mOsmol/kg H_2_O; PLA 287 ± 4 mOsmol/kg H_2_O; *P* = 0.773) and urine specific gravity (CIT 1.010 ± 0.007; PLA 1.013 ± 0.006; *P* = 0.231) were not different between trials.

#### Environmental conditions

Temperature (CIT 32.4 ± 0.4 °C; PLA 32.2 ± 0.4 °C, *P* = 0.398), relative humidity (CIT 52.5 ± 2.1%; PLA 53.0 ± 2.6%, *P* = 0.377) and facing windspeed (CIT 8.5 ± 0.9 km/h; PLA 8.7 ± 0.5 km/h, *P* = 0.110) were not different between trials.

#### Plasma L-citrulline and L-arginine

Plasma L-citrulline and L-arginine had main effects of time (*P* ≤ 0.016) and trial (*P* ≤ 0.017), and interaction effects (*P* ≤ 0.019), where both were greater with CIT. Post hoc tests revealed greater plasma L-citrulline (*P* ≤ 0.016; *g* = 1.02–1.12; Table 1) and L-arginine at all time points in CIT (*P* ≤ 0.024; *g* = 1.30–1.70; Table 1).

#### Running performance

**Fig. 1:**
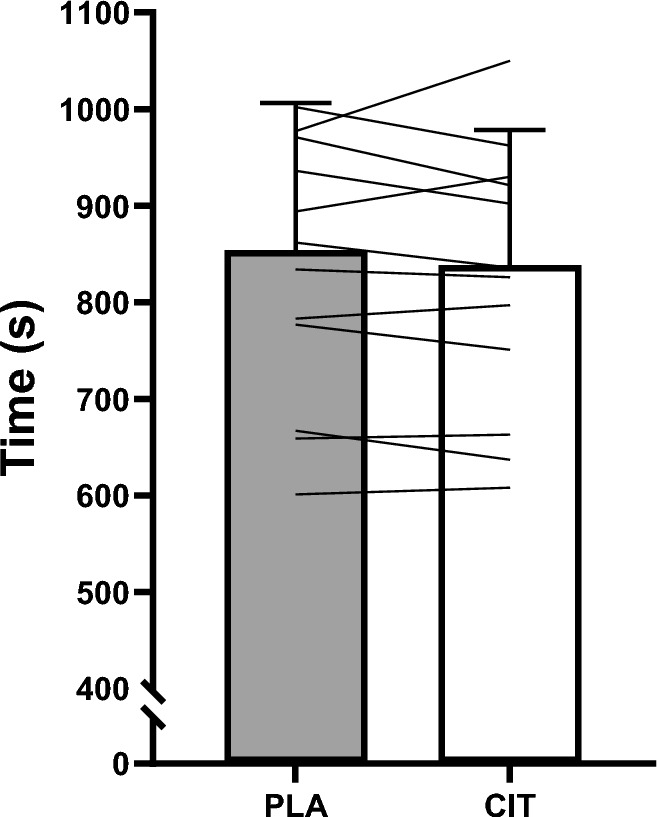
3 km time trial performance time for L-citrulline and placebo conditions. Bars indicate group mean, error bars indicate group standard deviation and lines represent individual participants

Time to complete the 3 km time trial performance was not different between conditions (*P* = 0.252; *g* = 0.109; Fig. 1). There was no presence of a trial order effect (Trial 1: 850 ± 154 s; Trial 2: 839 ± 140 s; *P* = 0.437; *g* = 0.07).

#### Hydration measures

**Fig. 2:**
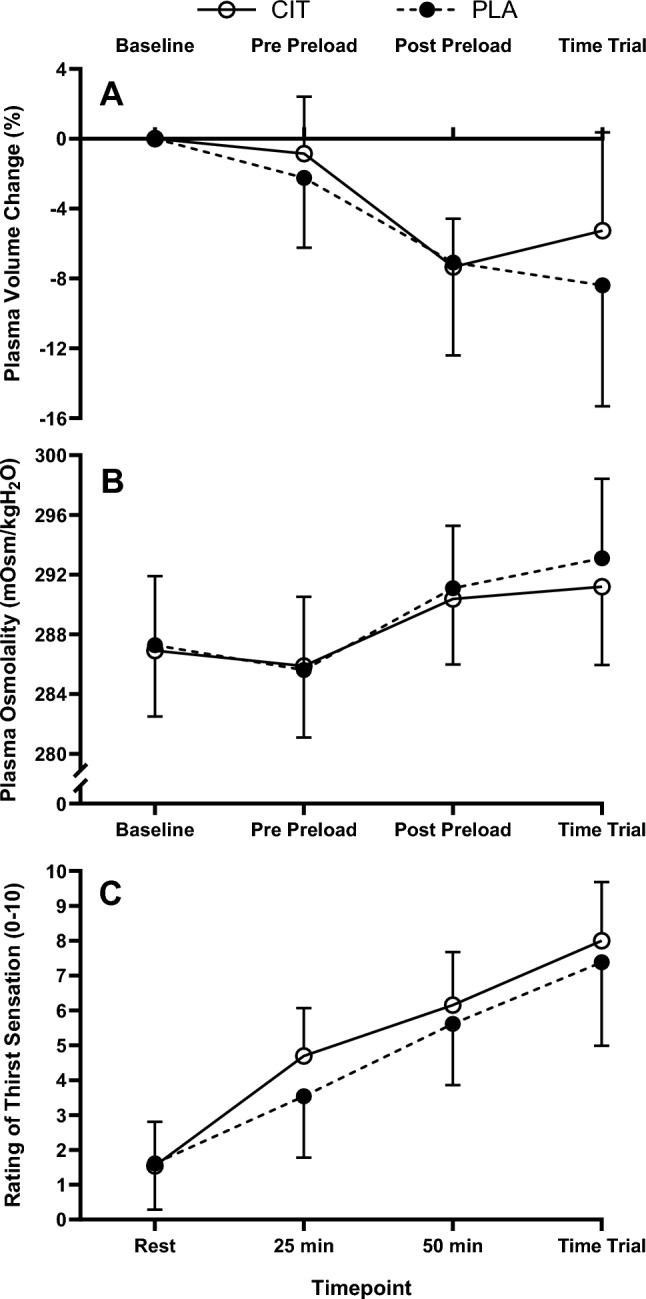
Plasma volume change (**A**), plasma osmolality (**B**), rating of thirst sensation (**C**) during the preload and time trial for L-citrulline and placebo trials

Change in body mass relative to baseline was not different between trials post-preload (CIT −1.5 ± 0.2%; PLA −1.5 ± 0.3%; *P* = 0.883;) or post-time trial (CIT −2.0 ± 0.2%; PLA −2.0 ± 0.4%; *P* = 0.945;). Plasma volume decreased, whilst plasma osmolality increased over time (*P* < 0.001), but were not different between conditions (interaction *P* ≥ 0.216, trial effect *P* ≥ 0.152; Fig. 2). Thirst sensation increased over time (*P* < 0.001) and had an effect of trial (*P* = 0.043) with greater mean thirst in CIT (CIT 5.1 ± 0.3; PLA 4.5 ± 0.4; *P* < 0.001), but there was no interaction effect (*P* = 0.109; Fig. 2). Whole body sweat rate was not different between trials following the preload (CIT 1.28 ± 0.19; PLA 1.29 ± 0.32 L/h; *P* = 0.807;) and local sweat rate was not different between trials (CIT 3.38 ± 2.18; PLA 3.55 ± 1.66 mg/cm^2^/min; *P* = 0.555;).

#### Subjective measures

**Fig. 3:**
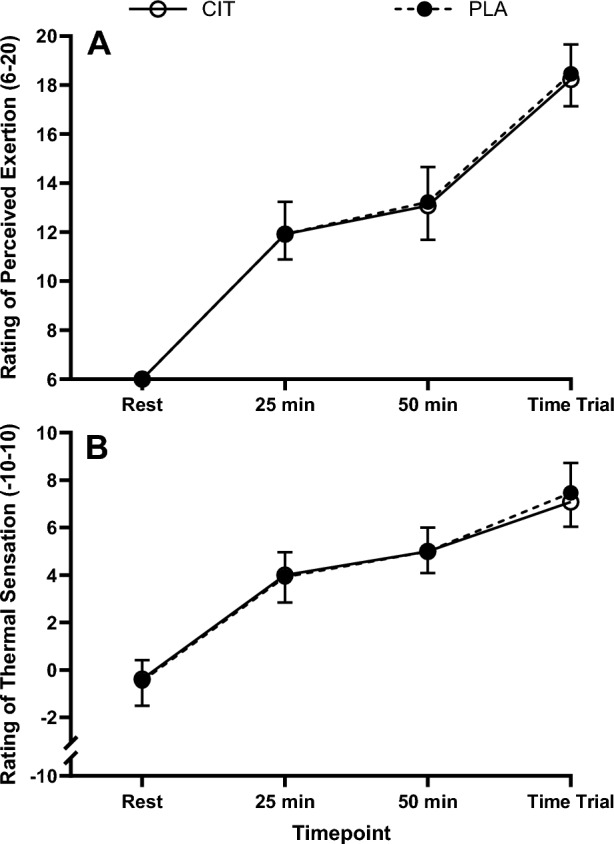
Rating of perceived exertion (**A**) and rating of thermal sensation (**B**) during the preload and time trial for L-citrulline and placebo trials

Subjective responses for RPE and thermal sensation increased over time (*P* ≤ 0.009) but were not different between trials (interaction *P* ≥ 0.610, trial effect *P* ≥ 0.628; Fig. 3).

#### Physiological measures

**Fig. 4:**
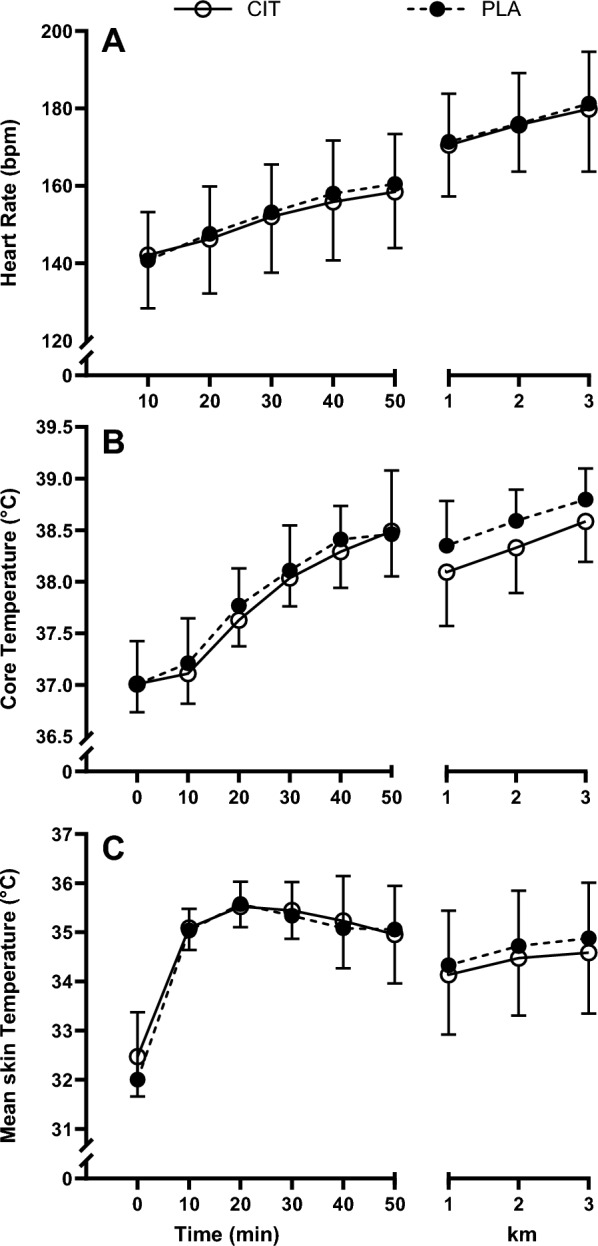
Heart rate (**A**), core temperature (**B**), and skin temperature (**C**) during the preload and time trial for L-citrulline and placebo trials

Heart rate, core temperature and skin temperature increased over the preload and TT (*P* < 0.001), but there were no trial (*P* ≥ 0.091) or interaction effects (*P* ≥ 0.270; Fig. 4).

**Fig. 5:**
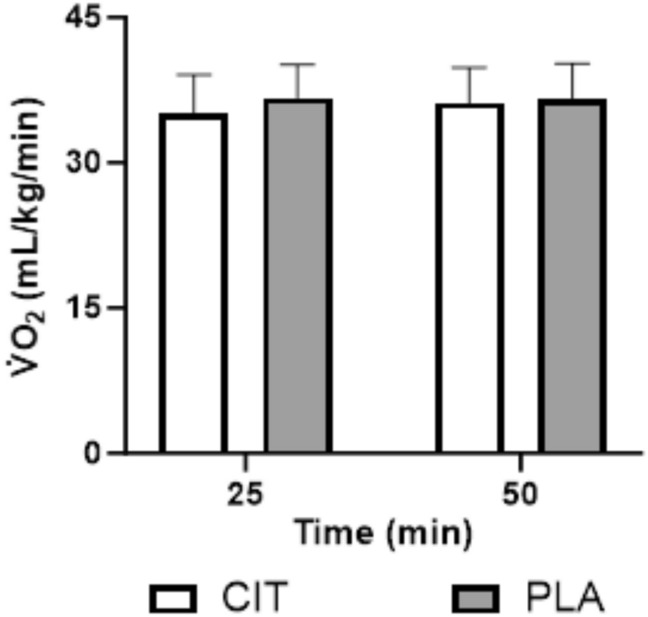
Oxygen uptake at 25 and 50 min of the preload for L-citrulline and placebo conditions

There was no effect of time for relative $\mathop {\text{V}}\limits^{.} {\text{O}}_{2}$ during the preload (*P* = 0.100) and, whilst there was no effect of trial (*P* = 0.160), there was an interaction effect for relative $\mathop {\text{V}}\limits^{.} {\text{O}}_{2}$ (*P* = 0.028), however, *post-hoc *tests did not reveal a difference at 25 min (*P* = 0.079) or 50 min (*P* = 0.476) (Fig. 5).

#### Gastrointestinal measures

**Fig. 6:**
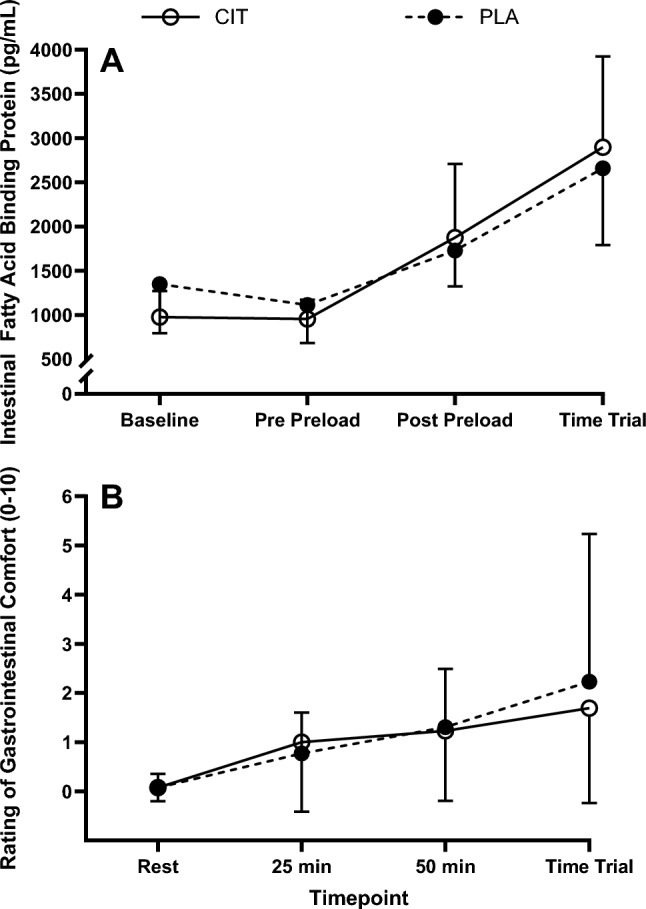
Intestinal fatty acid binding protein (**A**) and gastrointestinal comfort (**B**) during the preload and time trial for L-citrulline and placebo trials

Intestinal fatty acid binding protein increased following exercise (*P* < 0.001), but there were no trial (*P* ≥ 0.818) or interaction (*P* ≥ 0.260) effects (Fig. 6). Gastrointestinal comfort worsened over time (*P* = 0.009), but there were no trial (*P* ≥ 0.748) or interaction effects (*P* ≥ 0.531; Fig. 6).

## Discussion

In contrast to our hypothesis, 7 days of L-citrulline supplementation did not improve 3 km time trial performance. Moreover, although participants experienced mild hypohydration and had increased GI damage following exercise in the heat, no differences between L-citrulline and placebo were observed in thermoregulatory or GI responses. These findings were present despite the raised plasma L-arginine levels observed prior to exercise following 7 days supplementation with L-citrulline.

Whilst previous work has reported L-citrulline supplementation enhanced endurance cycling performance in a temperate environment in healthy volunteers (Bailey et al. 2015), the present study did not find a performance improvement from L-citrulline supplementation in the heat, even though exercise was performed at a similar intensity domain (~ 600–800 s) and following similar supplementation regimens. Indeed, the current study employed a prolonged steady-state preload prior to the time trial in the heat, which may provide context to the contrasting performance outcomes.

The purported mechanism for enhanced thermoregulation and potential improvement in endurance performance following L-citrulline supplementation is increased NO bioavailability. However, prolonged heat exposure has been shown to independently increase plasma nitrite (a marker of NO; Steward et al. 2024), which could have diminished the potential for L-citrulline to yield positive ergogenic effects as eNOS activity may have reached maximal stimulation. Although, as plasma nitrite was not measured in the current study, it is difficult to determine this as a conclusive factor in the study outcome.

Previously, thermoregulatory benefits of increased L-arginine bioavailability have been shown during running exercise in rodents (Wanner et al. 2015) and at rest in broilers (Uyanga et al. 2021) with a reduced body temperature under thermal strain compared to placebo controls. Indeed, animals have different modalities of heat loss compared to humans [rodents and broilers primarily lose heat from the respiratory tract (Hankenson et al. 2018)], so these results do not appear to translate to humans, where evaporative heat loss is a far more important mechanism. Additionally, L-citrulline is ineffective at increasing muscle oxygenation in healthy individuals (Theodorou et al. 2021), whilst studies have reported that supplementation with dietary nitrate (an alternative method to increase NO bioavailability) in humans does not improve thermoregulation or performance in the heat (≥ 30.0 °C) compared to placebo (Amano et al. 2018; Kent et al. 2018; Smith et al. 2019; Fowler et al. 2021). Taken together, the results of the present study and previous studies suggest that nutritional modulation of NO bioavailability is unlikely to beneficially alter thermoregulation or endurance performance in humans in the heat.

High sweat rates are often synonymous with exercise in the heat due to the need to dissipate excess heat from the body and therefore, hypohydration is likely to accrue with prolonged running, particularly as runners may find it difficult to carry and consume the fluid necessary to replace sweat losses (Rollo et al. 2012; Dion et al. 2013). Running, at least at high-intensity, reduces gastric emptying rates and can cause GI discomfort (Leiper et al. 2001), which can reduce volitional fluid intake and induce greater hypohydration. This body water deficit is likely to exacerbate the hyperthermic induced vascular strain and inflict greater reductions in plasma volume and vascular function, which it was hypothesised L-citrulline might attenuate, although in the current study, this did not appear to be the case. Similar to our findings, Amano et al. (2018) showed that increasing plasma NO may not alter cutaneous vascular and sweating responses during exercise in the heat, with the mechanisms for this response currently unknown. Therefore, future studies should explore other strategies to mitigate the decline in performance under conditions of heat stress and hypohydration.

Gastrointestinal blood flow is significantly reduced during exercise, especially when performed in hot environments where total blood volume is compromised (Costa et al. 2019). Subsequently, splanchnic perfusion is reduced which may lead to increased GI damage and reduced macronutrient uptake (Costa et al. 2019). A single pre-exercise dose of L-citrulline (10 g) has been shown to reduce gastrointestinal damage (via a decrease in the GI damage marker, I-FABP) following 60 min of cycling at ~ 70% Wmax (van Wijck et al. 2014). The attenuation of gastrointestinal damage was attributed to an increase in splanchnic microvascular perfusion and resultant reduction in enterocyte damage, possibly due to increases in NO-mediated microvascular dilation with greater L-arginine bioavailability. Conversely, whilst the current study observed an increase in L-arginine bioavailability, it found no change in I-FABP concentration with a comparable exercise-induced increase in I-FABP compared to van Wijck et al. (2014) (72% vs 67% in the current study). However, van Wijck et al. (2014) conducted exercise in a thermoneutral environment, where increases in I-FABP are lower than exercise in a hot environment (as used in the present study) (Sumi et al. 2024). Therefore, L-citrulline supplementation may have more capacity to attenuate GI damage whilst exercising in thermoneutral environments, which may explain the disparity in findings. None-the-less, the present study suggests that L-citrulline is unlikely to decrease GI damage augmented by hypohydrated running in the heat, where GI damage is most likely to have negative implications for the athlete (Costa et al. 2019).

Interestingly, the present study demonstrates the power of both acute and chronic L-citrulline supplementation to fully augment plasma L-arginine concentrations. Comparing the two trials demonstrates that the 6 days L-citrulline supplementation produced an ~ 65 µmol/L greater plasma L-arginine concentration at baseline, whilst the final dose produced a further ~ 65 µmol/L increase. Therefore, future studies wishing to maximise plasma L-arginine concentrations should consider using both acute and chronic L-citrulline supplementation. Although, if the primary objective is to improve endurance performance in the heat whilst hypohydrated, strategic L-citrulline supplementation of any form does not appear to be beneficial.

### Limitations

Indeed, there are limitations to the current study which future research could consider when conducting similar research studies. Firstly, the present study did not measure NO in the blood, so it is unclear if L-citrulline supplementation modified NO bioavailability directly, although plasma L-arginine was increased. Either way, the supplementation did not directly influence performance or thermoregulatory outcomes, suggesting it may not be a strategy worth exploring for exercise in the heat. Secondly, the present study did not prescribe or provide pre-trial diets and relied upon participant self-reported dietary control in the 24 h preceding experimental trials, although all participants confirmed they had weighed and recorded/replicated all foods and drinks upon arrival for each trial. However, this is a nuanced point and careful consideration needs to be given to participant motivation and compliance when choosing prescribed or habitual diets (Merrell et al. 2024). In the present study, the goal was to help ensure participants commenced trials in a similar metabolic state, meaning self-reported/controlled intake was likely adequate.

## Conclusion

In conclusion, 7 days of L-citrulline supplementation (6 g/day) did not alter 3 km running performance in trained runners in the heat, whilst in a hypohydrated state. Moreover, L-citrulline did not alter various physiological (i.e., heart rate, core, skin temperature, blood measures) or perceptual (i.e., RPE, thermal strain or gastrointestinal discomfort) responses during a 50 min preload or 3 km time trial, suggesting little rationale for its use in such situations.

## Supplementary Information

Below is the link to the electronic supplementary material.

Supplementary file1 (DOCX 17 KB)

## Data Availability

Data supporting these findings is available from the corresponding author upon reasonable request.

## Abbreviations

CIT: L-citrulline trial
PLA: Placebo trial
GI: Gastrointestinal
NO: Nitric oxide
eNOS: Endothelial nitric oxide synthase
