# Supplementary material for: 7 days of L-citrulline supplementation does not improve running performance in the heat whilst in a hypohydrated state
Source: Eur J Appl Physiol. 2024 Dec 19;125(5):1411–21. doi: 10.1007/s00421-024-05671-4 (PMC12055621; doi:10.1007/s00421-024-05671-4)
Supplement: Supplementary file 1 — Supplementary file1 (DOCX 17 KB) [file 421_2024_5671_MOESM1_ESM.docx]

*L-arginine/L-citrulline analysis.*

**Materials** L-arginine (ARG) and L-citrulline (CIT) hydrochloride were purchased from Alfa Aesar (Ward Hill, MA, USA). ARG hydrochloride-d7 and CIT-d6 were purchased from Toronto Research Chemicals (Toronto, Canada). Acetonitrile (MeCN), methanol (MeOH) and water were of LC-MS grade and purchased from VWR (Lutterworth, UK). Ammonium formate and formic acid (HCOOH) were purchased from Fisher Scientific (Loughborough, UK).

**Calibration and QC samples**

Stock solutions of ARG and CIT at 1 mM were prepared in 2% HCOOH and diluted accordingly to generate calibration standards at 10, 20, 50, 100, 200 and 333.33 µmol/L. All calibration experiments were calculated using a 1/x weighting and showed excellent linearity with calibration coefficients (r^2^) of >0.99. A quality control (QC) sample was created using a blank (baseline) plasma sample. Stock solutions of each internal standard (IS) were produced at 1.5 mM in 2% HCOOH and a working IS solution was created by diluting ARG-d7 and CIT-d6 in 2% HCOOH to a concentration of 150 µmol/L. All calibration and QC samples were processed as detailed below.

**Sample Preparation**

All samples were prepared as reported by Shin et al. (1). Fifty µL of each plasma sample, QC sample and calibration standard was mixed with 50 µL IS working solution and 50 µL water. To this, 850 µL of MeOH was added and the mixture vortexed followed by centrifugation at 20,000 *x*g for 15 min. One hundred µL of the supernatant was transferred to an autosampler vial, diluted with 900 µL of the starting mobile phase, and mixed thoroughly.

**LC-MS/MS**

Samples and standards were analysed by liquid chromatography-tandem mass spectrometry (LC-MS/MS) using an Acquity UPLC coupled to a Quattro Ultima triple quadrupole mass spectrometer (Waters Corp., Wilmslow, UK). Sample analyses were completed using an adapted protocol by Shin et al., (2019). An Aqcuity UPLC BEH HILIC column (130 Å, 1.7 μm, 2.1 mm × 100 mm, Waters) with an Acquity BEH HILIC VanGuard pre-column (130 Å, 1.7 μm, 2.1 mm × 5 mm, Waters) was used. Buffer A was 20 mM ammonium formate in 0.1% HCOOH and buffer B was 20 mM ammonium formate/0.1% HCOOH in 90% MeCN. An injection volume of 5 µL using a full loop setup and flow rate set at 300 µL/min, a column temperature of 30 °C, and an autosampler internal temperature of 10 °C was used. The gradient started with 10% buffer A for 3 min, rising linearly to 30% at 6 min, held for 1 min, and returned to starting conditions with an 8 min equilibration step. The total analysis time was 15 min. Multiple reaction monitoring was performed in positive ion mode ESI using the transitions detailed in Table S1 with ion source parameters for the mass spectrometer in Table S2. All analyses were performed using MassLynx (v4.1, Waters). Confirmation of analyte was achieved by multiple reaction monitoring (MRM) transition and retention time. Analyte peak areas, calibration statistics and sample concentrations were calculated using QuanLynx (Waters) by reporting the ratio of the peak area of the analyte to its corresponding internal standard. All concentrations were reported in micromoles per litre (µmol/L).

Table S1. Mass spectrometry parameters for multiple reaction monitoring of arginine, citrulline, and their respective labelled standards.

| **Compound** | **Q1 (*m/z*)** | **Q3 (*m/z*)** | **CE (V)** | **Dwell (ms)** |
| --- | --- | --- | --- | --- |
| Arginine | 175.1 | 70.0 | 20 | 100 |
| Arginine-d7 | 182.2 | 77.1 | 20 | 100 |
| Citrulline | 176.1 | 70.0 | 18 | 100 |
| Citrulline-d6 | 182.2 | 76.1 | 18 | 100 |

CE = collision energy

Table S2. Mass spectrometry source parameters for measurement of arginine, citrulline, and their respectively labelled standards.

| **Condition** | **Setting (unit)** |
| --- | --- |
| Capillary Voltage | 0.5 (kV) |
| Cone Voltage | 35 (V) |
| Source Temperature | 130 (°C) |
| Desolvation Temperature | 400 (°C) |
| Desolvation Gas Flow | 650 L/hr |
| Cone Gas Flow | 150 L/hr |
